# Spatial Frequency Effective for Increasing Perceived Glossiness by Contrast Enhancement

**DOI:** 10.3389/fpsyg.2021.625135

**Published:** 2021-02-05

**Authors:** Hiroaki Kiyokawa, Tomonori Tashiro, Yasuki Yamauchi, Takehiro Nagai

**Affiliations:** ^1^Department of Electrical Engineering and Informatics, Yamagata University, Yamagata, Japan; ^2^Japan Society for the Promotion of Science, Tokyo, Japan; ^3^Department of Informatics and Electronics, Yamagata University, Yamagata, Japan; ^4^Department of Information and Communications Engineering, Tokyo Institute of Technology, Yokohama, Japan

**Keywords:** material perception, glossiness, image manipulation, spatial frequency, psychophysics

## Abstract

It has been suggested that luminance edges in retinal images are potential cues for glossiness perception, particularly when the perception relies on low-luminance specular regions. However, a previous study has shown only statistical correlations between luminance edges and perceived glossiness, not their causal relations. Additionally, although specular components should be embedded at various spatial frequencies depending on the micro-roughness on the object surface, it is not well understood what spatial frequencies are essential for glossiness perception on objects with different micro-roughness. To address these issues, we examined the impact of a sub-band contrast enhancement on the perceived glossiness in the two conditions of stimuli: the Full condition where the stimulus had natural specular components and the Dark condition where it had specular components only in dark regions. Object images with various degrees of surface roughness were generated as stimuli, and their contrast was increased in various spatial-frequency sub-bands. The results indicate that the enhancement of the sub-band contrast can significantly increase perceived glossiness as expected. Furthermore, the effectiveness of each spatial frequency band depends on the surface roughness in the Full condition. However, effective spatial frequencies are constant at a middle spatial frequency regardless of the stimulus surface roughness in the Dark condition. These results suggest that, for glossiness perception, our visual system depends on specular-related information embedded in high spatial frequency components but may change the dependency on spatial frequency based on the surface luminance to be judged.

## Introduction

Human beings can effortlessly perceive the qualities of an object’s surface, such as its glossiness, at a single glance. The perception of surface qualities can be considered a brain function for estimating the object surface properties from retinal information. However, retinal images are created based on complicated interactions of object shapes, surface reflectance properties, and illumination environments. Thus, dissociating the surface reflectance properties from the shapes and illuminations by relying solely on the retinal images is a typical ill-posed problem. Instead, it has been reported that our visual system utilizes simple image features as heuristics to perceive different types of surface qualities without solving such ill-posed problems, as described below.

Glossiness is the surface quality that has been most frequently investigated. Previous research has typically focused on the roles of simple image features embedded in high-luminance regions, such as specular highlights, for glossiness perception. They have suggested the possible roles of simple image statistics, which mainly reflect the features of specular highlights, for glossiness perception (Nishida and Shinya, 1998; Fleming et al., 2003; Motoyoshi et al., 2007; Motoyoshi and Matoba, 2012; Wiebel et al., 2015). Motoyoshi et al. (2007) suggested that our visual system perceives the apparent surface gloss depending on the luminance histogram’s skewness of entire and/or spatial-frequency sub-band images, though numerous recent studies have claimed that a more sophisticated analysis of visual scenes, such as three-dimensional shapes of objects, are crucial for glossiness perception (Anderson and Kim, 2009; Kim et al., 2011, 2014; Marlow et al., 2011). Because the “tail” of the luminance histogram typically corresponds to specular highlights, such a claim suggests the importance of specular highlights or high luminance regions on the object surface for glossiness perception. This is consistent with previous studies supporting the importance of specular highlights in glossiness perception (Beck and Prazdny, 1981; Ferwerda et al., 2001; Marlow et al., 2012; Marlow and Anderson, 2013; van Assen et al., 2016; Di Cicco et al., 2019). For instance, Marlow et al. (2012) suggested that the coverage, contrast, sharpness, and depth of the specular highlights are informative predictors of human glossiness perception based on the results indicating that perceived gloss can be accurately predicted from such features, at least for their stimulus set.

In recent studies, it was proposed that low-luminance regions may also contain image cues for glossiness perception. Such image cues are different from those in the high-luminance regions. Kim et al. (2012) reported that not only bright regions, but also dark regions contribute to glossiness perception. They generated *dark gloss images*: First, they rendered images of specular objects and those of matte objects. Subsequently, by comparing these two images’ luminance values, they divided the specular image pixels into the bright and dark pixels. Finally, the bright pixels were replaced with pixels of the matte images. As a result, the specular highlights’ luminance values were lowered to those of the matte images using this image manipulation. Applying these images as stimuli, the researchers showed that observers perceived glossiness even from dark gloss images as well as normal specular images (we refer to this type of glossiness as *dark gloss*, hereafter). Their results indicate that there are different image cues for gloss perception from those derived from high-luminance regions such as luminance skewness (Motoyoshi et al., 2007) and highlight-related image features (Marlow et al., 2012).

What image features in low-luminance regions are cues for glossiness perception? Kim et al. (2016) compared the adaptation effects for glossiness perception on mirror-like objects between two types of adaptors: contour (luminance edge) adaptors and luminance-skewed texture adaptors. Their results showed that adaptation to the contour adaptors was much stronger than that by luminance-skewed texture adaptors, suggesting the importance of luminance edges for glossiness perception on certain classes of object images. Such luminance edges are also contained in low-luminance regions and not only in specular highlights. Similarly, Kiyokawa et al. (2019) reported strong correlations between the number of luminance edges and perceived glossiness, particularly on object images in which specular highlights do not seem very effective for glossiness perception. These findings suggest that luminance edges, in addition to highlight-related image features, are an essential glossiness cue, especially for dark gloss. Thus, we focus on the luminance edges’ detailed roles in high- and low-luminance regions for glossiness perception in this study.

However, the effective spatial frequency of the “luminance edges” for glossiness perception is poorly understood. For instance, Kim et al. (2016) claimed the importance of luminance edges of mirror-like reflections on glossiness. However, ordinary objects in the real world have micro-scale undulations, or roughness, on the surfaces in varying degrees. Thus, such a rough surface makes the reflected images on the object surface much blurrier. As a result, the effective spatial frequency components for glossiness perception may also change with these surface properties.

Nevertheless, previous studies have examined the effectiveness of only high-frequency edges extracted using Laplacian filters (Kim et al., 2016; Kiyokawa et al., 2019), not that of such blurred edges.Boyadzhiev et al. (2015) demonstrated that impressions of different types of surface qualities, such as glossiness and apparent aging, can be controlled by selectively modifying the coefficients of several components included in higher and lower spatial frequency sub-bands. However, they focused on the roles of just the two sub-bands: lower and higher sub-bands. In summary, the causal relationship between different spatial frequency bands and glossiness perception on objects with a variety of surface roughness has not been directly investigated.

Moreover, luminance ranges in object images may interact with spatial frequencies that are effective for perceived glossiness. Kiyokawa et al. (2019) demonstrated that positive correlations between high spatial frequency components and perceived glossiness increased as the specular highlights’ impact on perceived glossiness decreased. In other words, it depended on the luminance levels of surfaces used for glossiness judgments to what extent the high spatial frequency components are effective for glossiness perception. Even the contrast sensitivity functions (CSFs) are known to change with background luminance (Campbell and Robson, 1968; Kim and Kim, 2010; Wuerger et al., 2020). Therefore, CSFs may induce the luminance dependency of informative spatial frequencies for glossiness. Thus, it is also an important issue to investigate such interactions between effective spatial frequency and surface luminance ranges used for glossiness judgment.

This study aims to elucidate (1) the causal relationship between sub-band contrasts in different spatial frequencies and glossiness perception. Additionally, we investigate (2) the relation of such sub-band contrast effect with surface roughness and (3) that with luminance levels of regions used for perception. In our psychophysical experiments, we investigate whether enhancement of contrast in sub-band spatial frequency can increase perceived glossiness for different stimuli. We find that enhancing the contrast in the high spatial frequency sub-band can increase perceived glossiness. Generally, the effective spatial frequency strongly depends on the surface roughness in the stimuli with high-luminance and low-luminance specular components. However, only for stimuli whose specular highlights are replaced with matte object images (i.e., dark gloss images), the effective spatial frequency is constant regardless of the surface roughness and close to the peak frequency of the luminance CSF. Our results suggest that high spatial frequency components, such as luminance edges, are cues for glossiness perception, and strategies in the visual system change depending on the luminance level of regions on which the visual system relies on perceived glossiness.

## Experiment 1

One of this study’s objectives is to check whether the effectiveness of the sub-band contrast on glossiness perception depends on the luminance levels of image regions used for glossiness perception, as suggested by Kiyokawa et al. (2019). The impact from high-luminance regions (specular highlights) can be quantified using an index called *highlight dependency* (*HD*) of glossiness. Kiyokawa et al. (2019) defined *HD* as the difference in glossiness between stimuli with and without specular highlights. To quantify *HD* for stimulus images to be used in Experiment 2, we measured perceived glossiness on these stimulus images before Experiment 2.

Further, by measuring the stimulus images’ glossiness, the correlations between perceived glossiness and sub-band contrast can be examined. This is interpreted as preliminary results showing the impacts of the sub-band contrast on perceived glossiness.

### Materials and Methods

#### Observers

Five males and four females, including one of the authors (HK), participated in Experiment 1 as observers. All observers had normal or corrected-to-normal visual acuity. All observers, except the author, were unaware of the purpose of the experiment. All experimental procedures were approved by the ethical committee of the Faculty of Engineering, Yamagata University, and followed the Code of Ethics of the World Medical Association (Declaration of Helsinki). Written informed consent was obtained from all observers.

#### Apparatus

The stimuli were presented on an LCD monitor (27-inch ColorEdge CX271-CN, EIZO, Japan, 2,560 × 1,440 pixels) in an dark room. All experimental procedures were controlled using MATLAB R2014b (MathWorks, United States) and Psychtoolbox 3 (Brainard, 1997) on a personal computer (Vostro 3900, Dell; Intel Core i5-4460; GeForce GTX 745; Ubuntu 14.04 LTS). The monitor’s gamma properties and spectral distributions were measured using a colorimeter (ColorCAL II, Cambridge Research Systems, United Kingdom) and a spectral photometer (SR-3, Topcon Technohouse Corporation, Japan), respectively, to calibrate the stimulus luminance and chromaticity. The observers’ responses were obtained using a mouse connected to the computer. Each observer’s head position was roughly fixed using a chin rest such that the viewing distance was maintained at approximately 57 cm from the monitor. The observers viewed the stimuli binocularly.

#### Stimuli

**FIGURE 1:**
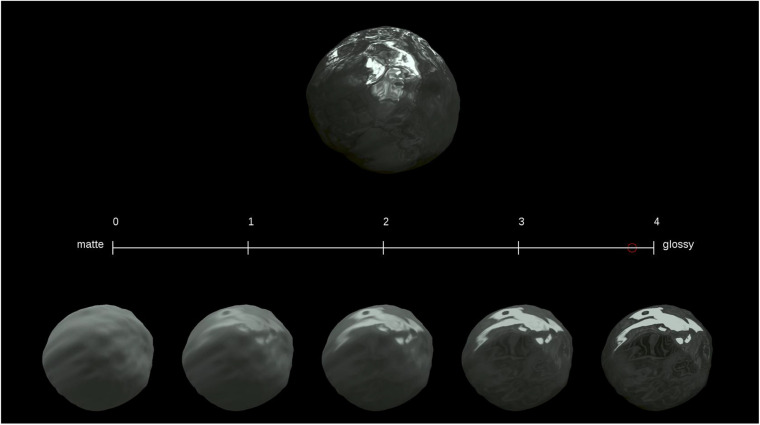
Stimuli applied during Experiment 1. The upper object image is a test stimulus, the lower five object images are reference stimuli, and the middle line is an evaluation axis.

The stimuli, shown in Figure 1, consisted of a test stimulus, an evaluation axis, and five reference stimuli. The test stimulus was presented at the upper screen area, and the evaluation axis with the reference stimuli was shown at the lower area of the screen. During the experiment, the observers evaluated perceived glossiness on the test stimulus by referring to the reference stimuli.

**FIGURE 2:**
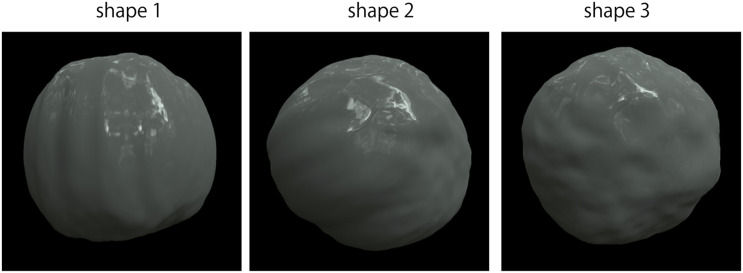
Object shapes.

The test stimulus was a computer graphics image, but its luminance was changed after rendering, as described in the next paragraph. The computer graphics images were created as follows. Blender 3D (ver. 2.79b; Blender Foundation, 2018) was used to model the stimulus objects’ shapes. The shape was created based on a UV sphere, a primitive sphere shape composed of horizontal and vertical meshes in Blender 3D. The UV sphere with 6,146 vertices was deformed into a potato-like shape using the displace function, a built-in function of Blender 3D, based on randomly created cloud pattern textures with a deformation strength of 0.25. The surfaces were smoothed using the built-in smoothing function. The three shapes shown in Figure 2 were made in this manner. The object images were rendered using Rendertoolbox3 (Heasly et al., 2014) in collaboration with Mitsuba renderer (Jakob, 2010). The test stimulus was created from a weighted linear addition of the images of a fully specular object and a fully matte object. Ward model (Ward, 1992) was adopted as the surface reflectance model of these objects. It has three physical parameters: specular reflectance ρ_*s*_, diffuse reflectance ρ_*d*_, and surface roughness α. Here, (ρ_*s*_,ρ_*d*_) of the fully specular object and fully matte object were (0.4, 0) and (0, 0.4), respectively. Then, a stimulus image was created according to the following equation:

(1) $$I_{\left(x,y\right)}=A\,I_{s\,\left(x,y\right)}+\left(1-A\right)\,I_{d\,\left(x,y\right)},$$

where *I* is the luminance of a given image pixel (*x*, *y*), *I*_*s*_ and *I*_*d*_ are the luminance of the fully specular and matte objects, respectively, and *A* is the specularity level. The images created by equation (1) are equivalent to images created under a parameter interpolated between (ρ_*s*_,ρ_*d*_) = (0.4, 0)–(0, 0.4). Furthermore, this equation was the same as that used in previous studies on dark gloss (Kim et al., 2012; Kiyokawa et al., 2019). In addition, *A* and α for the test stimuli were pairs of *A* = [0.2, 0.5, 0.9] and α = [0.01, 0.05, 0.1], leading to nine parameters in total. We show the nine images of different specularity levels *A* and roughness α in Supplementary Figure S1. All test images were rendered under either of two illumination maps: “Mossy Forest” and “Vintage Measuring Lab” at a resolution of 2K (2,048 × 1,024 pixels) obtained from a non-commercial repository of illumination map images (HDRI Heaven^1^). The illumination maps were employed to generate photo-realistic images as stimuli because previous studies demonstrated that object images look more photo-realistic by rendering them under illumination maps (Fleming et al., 2003; Kim et al., 2011; Motoyoshi and Matoba, 2012).

Their luminance was adjusted to create different types of test stimuli in their luminance conditions after rendering the object images. We had two conditions regarding object luminance manipulations, which are different in the presence or absence of specular highlights, as described by Kim et al. (2012) and Kiyokawa et al. (2019): the Full and Dark conditions, respectively (referred to as the *highlight conditions* hereafter). In the Full condition, the rendered images were used as stimulus images without modification. In the Dark condition, the luminance values of the rendered images were modified according to the following equations:

(2) $$\begin{array}{c} I_{D\,a\,r\,k\,\left(x,y\right)}=I_{d\,\left(x,y\right)}\;f\,o\,r\,I_{s\,\left(x,y\right)}>I_{d\,\left(x,y\right)}, \end{array}$$

(3) $$\begin{array}{c} I_{D\,a\,r\,k\,\left(x,y\right)}=I_{\left(x,y\right)}\;o\,t\,h\,e\,r\,w\,i\,s\,e, \end{array}$$

where *I*_*Dark*_ is the luminance in the Dark condition image for a given pixel (*x*,*y*). In these equations, first, the luminance values of the matte component [*I*_*d*_ in Eq. (1)] were compared with those of the corresponding specular components [*I*_*s*_ in Eq. (1)] in a pixel-by-pixel manner. Second, pixels with (*I*_*s*_ > *I*_*d*_) were identified as *highlight pixels*. Finally, the luminance values of all highlight pixels in the stimulus images [*I* in Eq. (1)] were replaced with those of a fully matte object image (*I*_*d*_). By applying this modification, the specular highlights’ luminance values were replaced by those of the corresponding pixels in the fully matte object images. Conversely, the other regions were preserved in the Dark condition. It should be noted that this definition of specular highlights is based on a simple luminance comparison between specular and matte objects and does not necessarily correspond to the regions of perceptual specular highlights. After creating the Full and Dark condition stimuli, the stimulus images were made achromatic [CIE1931 *xy* chromaticity, (*x*, *y*) = (0.313, 0.329)]. Further, the mean luminance was equalized to 9.9 cd/m^2^ across all stimuli to prevent observers from responding based simply on brightness not on glossiness. Pixels that exceeded the maximum (123.2 cd/m^2^) or minimum (0.3 cd/m^2^) luminance of the monitor were rounded to the maximum or minimum values. We generated 108 test stimuli in total (3 shapes × 3 specularity levels (*A*) × 3 surface roughness values (α) × 2 illumination maps × 2 highlight conditions). The generated test stimuli’s size was 800 × 800 pixels, and then the images were resampled to 400 × 400 pixels using the built-in anti-aliasing filter in MATLAB. This size corresponds to 7.4 × 7.4 degrees in terms of the visual angle.

The reference stimuli were also created in the same way as the Full condition test stimuli, except for the rendering parameters and illumination map. We created a new shape for the reference stimuli in the same fashion as the test stimuli. The rendering parameters applied for the five reference stimuli were (*A*,α) = (0.0, 0.1), (0.25, 0.075), (0.5, 0.05), (0.75, 0.025), and (1.0, 0.01). These parameters were arbitrarily determined such that perceived glossiness for the five stimuli was arranged at roughly equal intervals. “Uffizi Gallery” was used as the illumination map (Debevec, 1998). The reference stimuli had specular highlights as well as Full condition stimuli regardless of the test stimulus’s highlight condition. The generated reference stimulus images’ size was originally 600 × 600 pixels and then was resized to 300 × 300 pixels by the nearest neighbor method when presented. This corresponds to 5.5 × 5.5 degrees in terms of the visual angle.

#### Procedure

We adopted a simple rating task to measure the perceived glossiness of the stimuli. During each trial, the observer was asked to rate the test stimulus’s perceived glossiness on a 5-point scale (0–4) by moving the red circle along the evaluation axis using a mouse in reference to the reference stimuli. The rated value was defined as the glossiness score. The observation time was not restricted. All stimuli were rated once in random order during each session, leading to 108 trials. Each observer participated in four sessions.

### Results and Discussion

**FIGURE 3:**
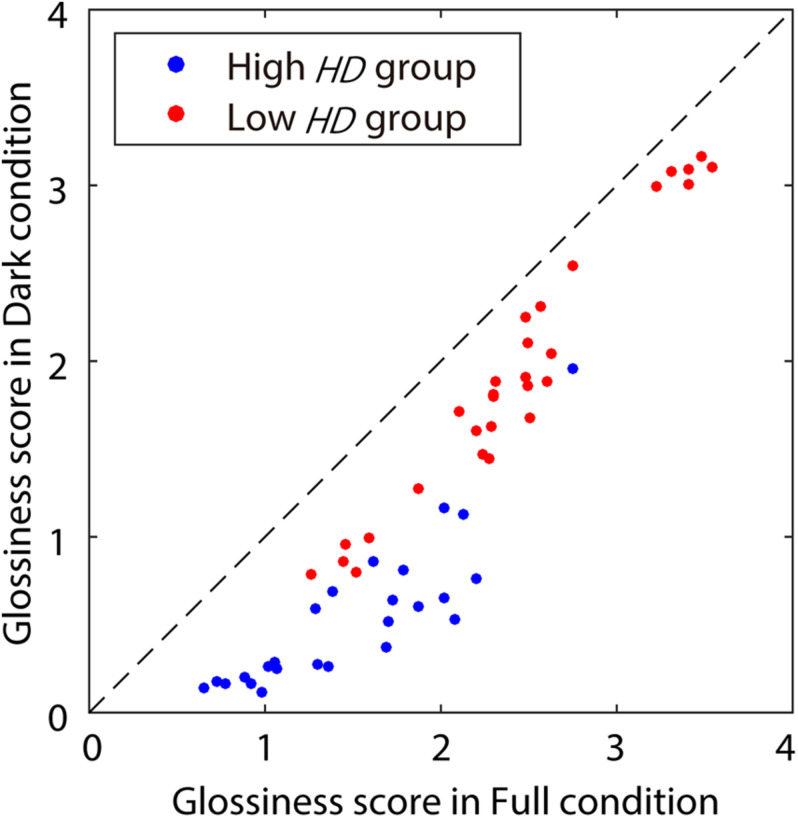
Glossiness scores measured during Experiment 1. The *x*- and *y*-axes show glossiness scores in Full and Dark conditions, respectively. The dot colors indicate the *HD* groups: blue for the high *HD* group (with significant differences between Dark and Full), and red for the low *HD* group (with no significant difference). The diagonal dashed line indicates the equal scores between the highlight conditions.

The glossiness scores are shown in Figure 3. Each plot point indicates glossiness scores averaged across four reputations and all observers for a stimulus. The glossiness scores tended to be slightly lower in the Dark condition than in the Full condition. This is intuitively plausible because the luminance values in the highlight pixels were lowered in Dark conditions. We conducted a *t*-test to check the statistical significance of the differences in glossiness scores between the Dark and Full conditions for each stimulus under the significance level of 0.05. In this testing procedure, the test was repeated 54 times (i.e., for 54 stimuli). Thus, according to the Bonferroni correction, the significance level was corrected to *p* < 0.0009 ( = 0.05 / 54 pairs). In Figure 3, the blue plots indicate stimuli with significant differences, and the red plots indicate stimuli with no significant differences. Approximately half of the stimuli had significantly lower glossiness for the Dark condition. Based on these results, we divided the stimuli into two groups: a high highlight dependency (high *HD*) group, which showed significantly lower glossiness for the Dark condition, and a low highlight dependency (low *HD*) group, which showed no significant differences between the highlight conditions. The number of stimuli was 25 in the high *HD* group and 29 in the low *HD* group.

We then calculated the correlation coefficients between the sub-band root-mean-square (RMS) contrasts in the various SF bands and glossiness scores for each combination of α, sub-band SF, and *HD* groups. Sub-band RMS contrasts were calculated from the stimulus images using two-dimensional (2D) finite impulse response (FIR) bandpass filters. They have a Gaussian window with *σ* of 1.0. The filter size was 300 × 300 pixels. Further, their bandpass was 1 octave; the filter’s central SF was 8, 16, 32, or 64 cycles per image (cpi), which correspond to 1.08, 2.16, 4.32, or 8.65 cycles per degree (cpd), respectively. It should be noted that one of the critical issues in glossiness perception is whether the underlying mechanisms are scale-variant or scale-invariant. For instance, scale-invariant mechanisms are mainly involved in object recognition (Karimi-Rouzbahani et al., 2017; Han et al., 2020), although it seems a higher-order visual function than glossiness perception. However, little is known about which scale-variant or -invariant mechanisms dominate glossiness perception. Therefore, we notate both “cpi” and “cpd” to represent spatial frequencies throughout this paper.

**FIGURE 4:**
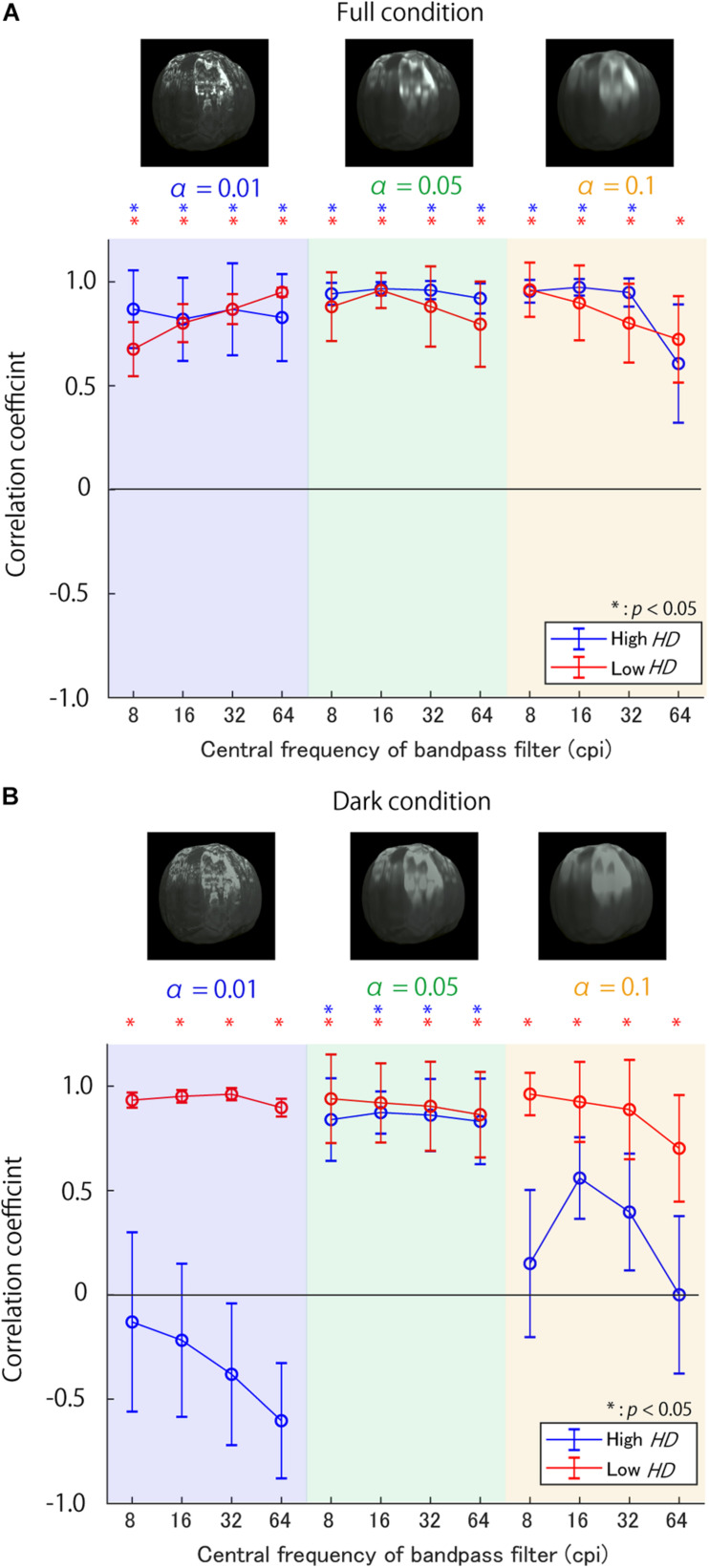
Correlation coefficients between sub-band contrast on various SFs and glossiness scores **(A)** in Full condition and **(B)** Dark condition. The background colors show the surface roughnessα, while the line colors denote the *HD* group. The asterisks indicate the conditions with significantly positive correlations. The error bars show ±1 standard error of means (SEM) of the correlation coefficients. The SEM was calculated using a bootstrap procedure; the bootstrapped correlation coefficients’ standard deviations were defined as SEMs. The object images above each figure are example stimuli in different αs.

The correlation coefficients are shown in Figure 4A for the Full condition and Figure 4B for the Dark condition. In the Full condition (Figure 4A), the sub-band RMS contrasts correlated well with the glossiness scores as a whole, although the trend along the SFs seems to depend moderately on α and the *HD* group. The correlations in the low *HD* group showed a moderate interaction between α and the SFs; the higher the value of α, the lower the SFs of the maximum correlation coefficients. By contrast, those in the high *HD* group did not exhibit such apparent effects of α. On the contrary, the Dark condition results (Figure 4B) showed different and complicated trends. First, the sub-band RMS contrasts correlated well with the glossiness scores in the low *HD* group regardless of α as in the Full condition. In contrast, the correlations were almost lost in the high *HD* group except for α of 0.05, suggesting the possibility that the sub-band contrast is effective only in the low *HD* group.

To test the statistical significance of the interactions between α and sub-band SFs, we performed a 3-way repeated measures ANOVA in each highlight condition. The factors were surface roughness, sub-band SF, and *HD*. The Mendoza’s multisample sphericity test did not guarantee the sphericity of surface roughness by a 5% significance level in the Dark condition. Therefore, we adjusted the degrees of freedom for the ANOVA. There was a significant interaction between surface roughness and target sub-band SF for both the highlight conditions [*F*_(__1.42, 12.82)_ = 11.65, *p* < 0.01 in the Dark condition; *F*_(__6, 54)_ = 24.57, *p* < 0.001 in the Full condition]. These results support the possibility that the effective SFs for glossiness perception gradually change with the surface roughness α as expected.

Based on these results, we aim to examine further the causal effects of the contrast manipulation in high SF bands on glossiness perception in each α and the *HD* group in Experiment 2.

## Experiment 2

Experiment 1 demonstrated that the sub-band contrast correlated with perceived glossiness depending on surface roughness. In Experiment 2, we investigated (1) whether enhancing the contrast in various SF sub-bands can increase perceived glossiness, (2) the most influential SF for manipulating the glossiness and its relation to the surface roughnessα, and (3) whether the most effective SF depends on the luminance levels of the regions contributing to glossiness perception. To achieve these objectives, we measured perceived glossiness on object images with different values of α after enhancing the contrast in various SFs. Moreover, regarding the third purpose of this experiment, we focused on two kinds of stimulus parameters. The first was *HD*, which represents the relative importance of luminance ranges in image regions for glossiness judgments, as suggested by Kiyokawa et al. (2019). The second one was the highlight condition (the Full and Dark conditions), which directly controlled high-luminance regions’ contributions. In the experiment, we compared the impact of sub-band contrast between high and low *HD* groups, and Full and Dark stimuli.

### Materials and Methods

#### Observers and Apparatus

Nine observers (seven males and two females) and one of the authors (HK) participated in Experiment 2. All observers, except for two of the males, also participated in Experiment 1. All observers, except the author, were unaware of the purpose of the experiment. All observers had normal or corrected-to-normal visual acuity. All experimental procedures were approved by the ethical committee of the Faculty of Engineering, Yamagata University, and followed the Code of Ethics of the World Medical Association (Declaration of Helsinki). Written informed consent was obtained from all observers.

The same apparatus was used as for Experiment 1.

#### Stimuli

**FIGURE 5:**
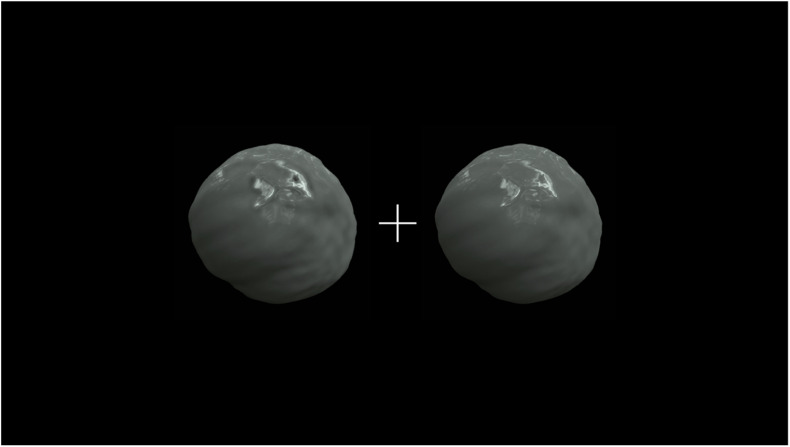
Stimulus in Experiment 2. The size of each object image, including the black background, was 400 × 400 pixels (visual angle of 7.4 × 7.4°).

**FIGURE 6:**
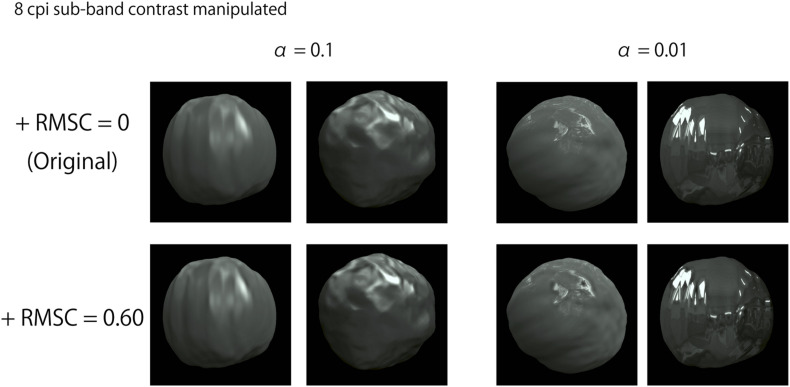
Example images with 8 cpi (1.08 cpd) contrast enhancement. “+RMSC” in the figure indicates an increased RMS contrast value.

An example stimulus is shown in Figure 5. Two object images were presented side-by-side. Each was created by manipulating the RMS contrast of a specific SF on a common object image used in Experiment 1 (one of the 108 images). The contrast of the image was manipulated based on the FIR bandpass filters’ sub-band images identical to those used in Experiment 1. First, we created a “baseline sub-band image” in one of the sub-band images (the central SF was 8, 16, 32, or 64 cpi. This is called *the target SF*), and then multiplied it with a coefficient for its RMS contrast to be 1. The baseline sub-band image was further multiplied with a coefficient of 0.00, 0.15, 0.30, 0.45, or 0.60. Finally, the luminance of the multiplied baseline sub-band image was added to the original image. For instance, for an object image with an original RMS contrast of 0.3 in the target SF, the RMS contrast after applying this procedure was 0.30, 0.45, 0.60, 0.75, or 0.90. The increased magnitudes in the RMS contrast were equalized across all stimuli and SFs, regardless of the original sub-band contrast. After the contrast enhancement, the pixels’ luminance that exceeded the monitor gamut was rounded to the monitor’s maximum or minimum luminance. Some images with 8 cpi (1.08 cpd) contrast enhancement are shown in Figure 6. The images with contrast enhancement in the other SFs are shown in Supplementary Figures S2–S4.

#### Procedure

The perceived glossiness was measured using the Thurstone’s paired comparison procedure. During each trial, the observer was presented with a stimulus—the two images differed only in the contrast enhancement strengths. The observers judged which image seemed glossier in a two-alternative forced-choice (2AFC) manner. They observed the stimulus freely until they were satisfied. The number of image pairs was 4,320 [ = $\left(\begin{array}{c} 5\\ 2 \end{array}\right)$ contrast pairs × 4 SFs × 54 objects × 2 highlight conditions]. The image pairs were presented in random order, and each observer responded only once per pair. These trials were conducted separately in eight sessions, each of which was composed of 540 trials.

### Results and Discussion

**FIGURE 7:**
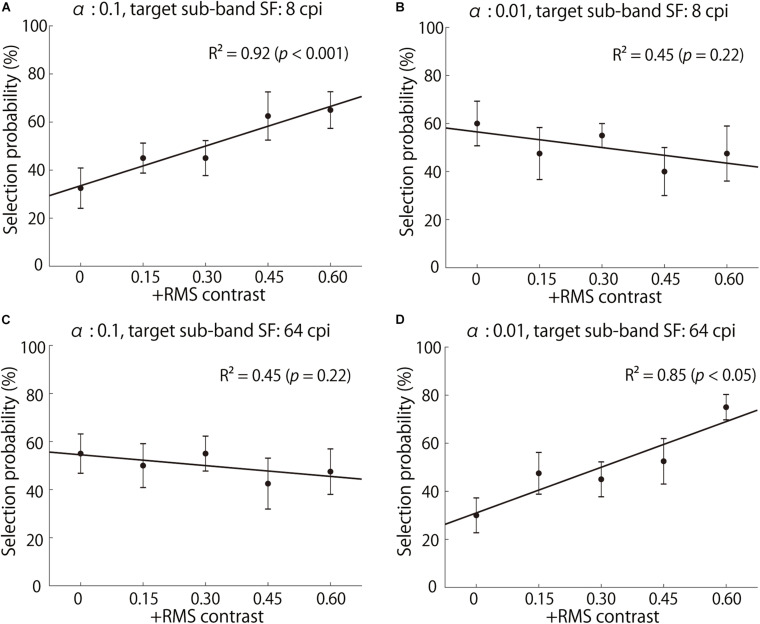
Selection probabilities averaged across observers. **(A,B)** show the results of 8 cpi (1.08 cpd) contrast-enhanced stimuli with α of 0.1 and 0.01, respectively. **(C,D)** show the results of 64 cpi (8.65 cpd) contrast-enhanced stimuli with α of 0.1 and 0.01, respectively. The *x*-axis indicates the increased RMS contrast, and the *y*-axis indicates a “glossier” selection probability. The solid lines represent the linear regression lines.

Figure 7 shows the “glossier” selection probability averaged across all observers for stimuli with 8 and 64 cpi contrast manipulation for two object images with αof 0.1 and 0.01. The contrast enhancement in 8 cpi (1.08 cpd) seemed to increase perceived glossiness on the object image with α of 0.1 (Figure 7A), but not on that with α of 0.01 (Figure 7B). On the other hand, the 64 cpi (8.65 cpd) contrast manipulation increased perceived glossiness on the object with α of 0.01 (Figure 7D), but not on the object image with α of 0.1 (Figure 7C). These results indicate that the enhancement of the sub-band contrast can increase perceived glossiness. However, the effectiveness of this image manipulation depends on the interaction between the target SF and surface roughness α.

To evaluate the contrast enhancement effectiveness quantitatively, we conducted linear regression analysis for each combination of α and target SF. In this analysis, the slopes of the regression, such as those in Figure 7, reflect the contrast enhancement’s effectiveness on glossiness. A regression analysis was conducted for the selection probability averaged across observers on each of the 432 images: 108 original images (3 shapes, 3 specularities, 3 α values, 2 illumination maps, and 2 highlight conditions) × 4 SFs. The regression slopes were averaged across the stimulus parameters other than α and target SF in each *HD* group (25 and 29 images in high and low *HD* groups).

**FIGURE 8:**
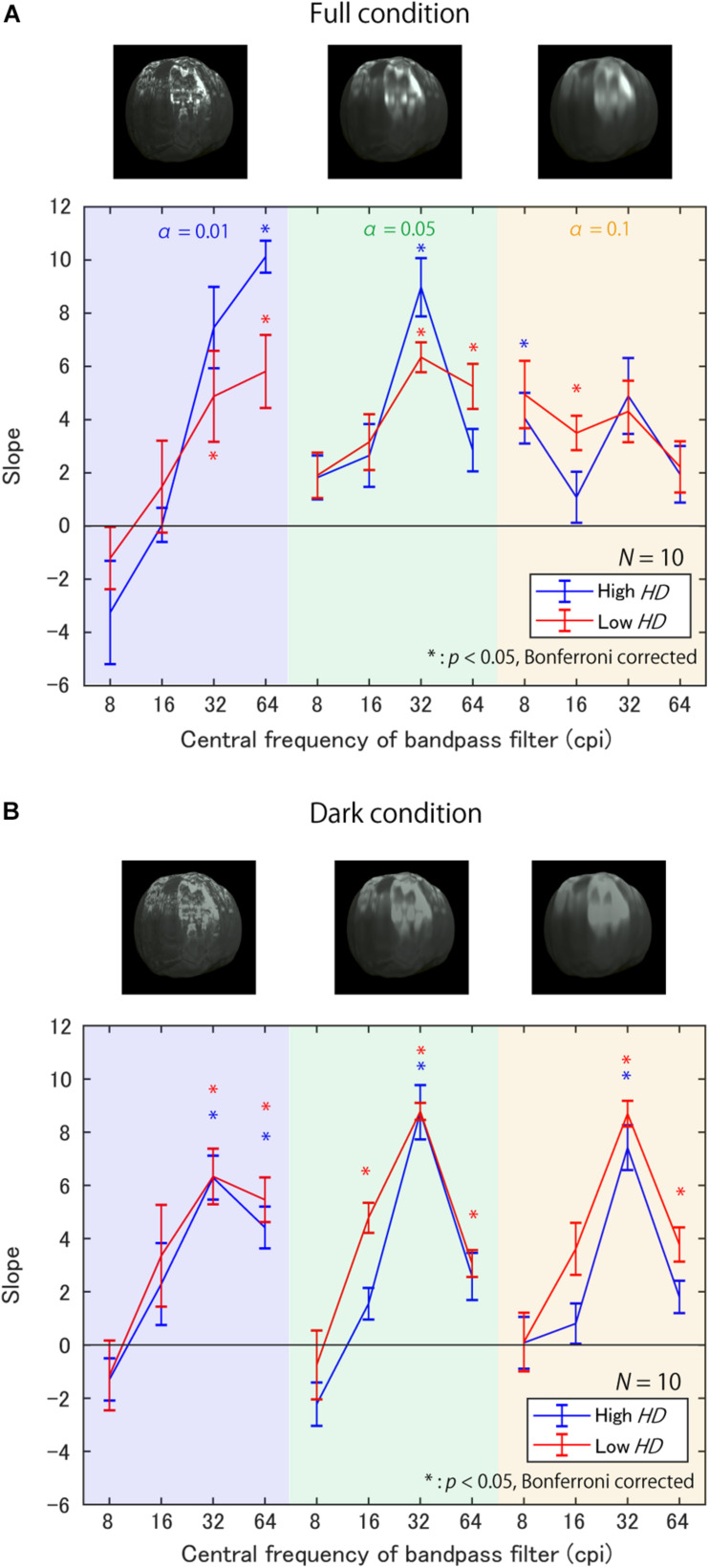
Slopes of regression lines between contrast enhancement strength and selection probability **(A)** in Full condition and **(B)** in Dark condition. The background colors represent α. Line colors denote the *HD* groups. Error bars denote ±1 *SEM* of the object images. Asterisks represent significantly positive slopes.

Figure 8A shows the slopes of the regression lines for each triad of α, target SF, and *HD* group in the Full condition. First, most of the slopes are positive, indicating that the sub-band contrast enhancement increased perceived glossiness, as expected. However, the SFs with maximum slopes vastly differed across surface roughness α; the SF with the maximum slope was 64 cpi (8.65 cpd) for α of 0.01 in both *HD* groups, but the maximum slope SF seems to decrease with an increase in α. These trends indicate that the effective SFs for the glossiness enhancement strongly depends on the fact that the higher the value of α, the lower the effective SF for a glossiness modulation. Conversely, the general trends do not differ between the high and low *HD* groups, except for the case of 64 cpi (8.65 cpd) and α of 0.01.

Figure 8B shows the slopes in the Dark condition. Again, most of the slopes are positive, suggesting the impact of the sub-band contrast enhancement. However, the SF’s effects seem to differ from the Full condition; the slopes are maximum at 32 cpi (4.32 cpd) regardless of α.

To test the statistical significance of these trends, we conducted two types of statistical hypothesis testing. The first was a one-sample *t*-test with a Bonferroni correction to check whether the slopes were significantly positive for each condition. The test results are summarized in Tables 1, 3 for the high and low *HD* groups in the Full condition, and Tables 2, 4 in the Dark condition, respectively.

**TABLE 1:** *p*-values of one-sample *t*-test to check whether the slopes were significantly positive in the high *HD* group and Full condition.

α	Target SF (cpi)	*t*-value	*p-*value	Effect size (|C⁢o⁢h⁢e⁢n⁢s′⁢⁢d|)
0.01	8	*t*_5_ = −1.68	0.1547	0.74
	16	*t*_5_ = 0.07	0.9506	0.03
	32	*t*_5_ = 4.88	0.0046	2.20
	**64**	***t*_5_ = 16.84**	**<0.0001**	**7.43**
0.05	8	*t*_9_ = 2.21	0.0543	0.73
	16	*t*_9_ = 2.24	0.0516	0.74
	**32**	***t*_9_ = 8.20**	**<0.0001**	**2.72**
	64	*t*_9_ = 3.58	0.0060	1.19
0.1	**8**	***t*_8_ = 4.26**	**0.0028**	**1.50**
	16	*t*_8_ = 1.13	0.2919	0.40
	32	*t*_8_ = 3.43	0.0089	1.21
	64	*t*_8_ = 1.83	0.1054	0.64

**The adjusted significance level was 0.0042 ( = 0.05/12). The conditions typed in bold fonts represent those with statistical significance.**

**TABLE 2:** *p*-values of one-sample *t*-test to check whether the slopes were significantly positive in the high *HD* group and Dark condition.

α	Target SF (cpi)	*t*-value	*p-*value	Effect size (|C⁢o⁢h⁢e⁢n⁢s′⁢⁢d|)
0.01	8	*t*_5_ = −1.63	0.1648	0.72
	16	*t*_5_ = 1.49	0.1963	0.66
	**32**	***t*_5_ = 7.62**	**0.0006**	**3.40**
	**64**	***t*_5_ = 5.63**	**0.0024**	**2.49**
0.05	8	*t*_9_ = −2.73	0.0232	0.90
	16	*t*_9_ = 2.60	0.0287	0.86
	**32**	***t*_9_ = 8.58**	**<0.0001**	**2.85**
	64	*t*_9_ = 2.90	0.0175	0.96
0.1	8	*t*_8_ = 0.09	0.9340	0.03
	16	*t*_8_ = 1.06	0.3181	0.37
	**32**	***t*_8_ = 8.79**	**<0.0001**	**3.09**
	64	*t*_8_ = 2.96	0.0180	1.04

*The adjusted significance level was 0.0042 (= 0.05/12). The condition typed in bold fonts represent those with statistical significance.*

**TABLE 3:** *p*-values of one-sample *t*-test to check whether the slopes were significantly positive in the low *HD* group in Full condition.

α	Target SF (cpi)	*t*-value	*p-*value	Effect size (|C⁢o⁢h⁢e⁢n⁢s′⁢⁢d|)
0.01	8	*t*_11_ = −1.46	0.1721	0.44
	16	*t*_11_ = 1.21	0.2508	0.36
	**32**	***t*_11_ = 4.02**	**0.0020**	**1.21**
	**64**	***t*_11_ = 5.99**	**<0.0001**	**1.80**
0.05	8	*t*_7_ = 2.00	0.0853	0.75
	16	*t*_7_ = 2.69	0.0309	1.01
	**32**	***t*_7_ = 10.09**	**<0.0001**	**3.79**
	**64**	***t*_7_ = 5.56**	**<0.0001**	**2.08**
0.1	8	*t*_8_ = 3.90	0.0045	1.37
	**16**	***t*_8_ = 5.42**	**0.0006**	**1.90**
	32	*t*_8_ = 3.73	0.0058	1.31
	64	*t*_8_ = 2.31	0.0496	0.81

*The adjusted significance level was 0.0042 (= 0.05/12). The condition typed in bold fonts represent those with statistical significance.*

**TABLE 4:** *p*-values of one-sample *t*-test to check whether the slopes were significantly positive in the low *HD* group in Dark condition.

α	Target SF (cpi)	*t*-value	*p-*value	Effect size (|C⁢o⁢h⁢e⁢n⁢s′⁢⁢d|)
0.01	8	*t*_11_ = −1.23	0.2427	0.37
	16	*t*_11_ = 2.48	0.0308	0.74
	**32**	***t*_11_ = 8.57**	**<0.0001**	**2.58**
	**64**	***t*_11_ = 9.21**	**<0.0001**	**2.77**
0.05	8	*t*_7_ = −0.52	0.6198	0.19
	**16**	***t*_7_ = 7.58**	**0.0001**	**2.84**
	**32**	***t*_7_ = 24.7**	**<0.0001**	**9.28**
	**64**	***t*_7_ = 5.43**	**<0.0001**	**2.03**
0.1	8	*t*_8_ = 0.10	0.9224	0.04
	16	*t*_8_ = 3.69	0.0061	1.30
	**32**	***t*_8_ = 18.0**	**<0.0001**	**6.34**
	**64**	***t*_8_ = 5.85**	**<0.0001**	**2.05**

*The adjusted significance level was 0.0042 (= 0.05/12). The condition typed in bold fonts represent those with statistical significance.*

In the Full condition, slopes are significantly positive only at high SFs for small α [e.g., 64 cpi (8.65 cpd) for α of 0.01 in Table 1], while significantly positive only at low SFs for large α (e.g., 8 cpi for α of 0.1 in Table 1), as expected from Figure 8A. On the other hand, in the Dark condition, significantly positive slopes always appeared only around 32 cpi (4.32 cpd) regardless of α. These general positive slopes suggest a causal relationship between the SF contrast and glossiness perception in accordance with previous studies (Kim et al., 2016; Kiyokawa et al., 2019). These results suggest that effective SFs are different across the bright and dark regions on the object surfaces.

The second test was a three-way repeated-measures ANOVA, in which the dependent variable was regression slopes, and the factors wereα, target SF, and the *HD* group. Mainly in checking: (a) whether the effective target SFs for a glossiness modulation change with α; (b) whether there are differences between the *HD* groups. Before performing ANOVA, we tested each factor’s sphericity and their interactions using Mendoza’s sphericity test. The results showed that there was no guarantee of sphericity on some factors and their interactions. Thus, we adjusted their degrees of freedom in the ANOVA by Greenhouse-Geisser’s epsilon. The ANOVA results are shown in Tables 5, 6 in the Full and Dark conditions, respectively. In the Full condition (Table 5), the main effects of the SF and the interaction between α and SF are statistically significant. This significant interaction implies that SFs effective for a glossiness enhancement change with α. In contrast, we found no statistical significance in the HD group’s main effects and in the interaction between the *HD* group and other factors. These results suggest that *HD* did not strongly influence the sub-band contrast’s effectiveness on the perceived glossiness in the Full condition.

**TABLE 5:** Results of three-way repeated-measures ANOVA in Full condition.

Factors	*F*-value	*p*-value
**α**	*F*_(__1.75_, _8.76__)_ = 3.39	0.09
**SF**	***F*_(__1.96_, _9.78__)_ = 46.46**	**<0.001**
*HD* group	*F*_(__1_, _5)_ = 0.84	0.40
**α × SF**	***F*_(__1.97_, _9.84__)_ = 13.05**	**<0.01**
**α** × *HD* group	*F*_(__1.7_, _8.48__)_ = 0.40	0.65
SF × *HD* group	*F*_(__2.13_, _1__0.63__)_ = 1.07	0.38
**α** × SF × *HD* group	*F*_(__2.22_, _11.11__)_ = 1.61	0.24

**The bold conditions had statistical significance in the main effect or interaction.**

**TABLE 6:** Results of three-way repeated-measures ANOVA in Dark condition.

Factors	*F*-value	*p*-value
**α**	*F*_(__1.53_, _7.67__)_ = 0.16	0.80
**SF**	***F*_(__1.27_, _6.35__)_ = 71.43**	**<0.001**
**HD group**	***F*_(__1_, _5)_ = 7.28**	**<0.05**
**α** × SF	*F*_(__3.__52_, _17.59__)_ = 1.53	0.24
**α** × HD group	*F*_(__1.45_, _7.23__)_ = 0.83	0.43
SF × HD group	*F*_(__1.76_, _8.8__)_ = 0.24	0.76
**α** × SF × HD group	*F*_(__3.26_, _16.32__)_ = 0.32	0.82

*The bold conditions had statistical significance in the main effect or interaction.*

On the other hand, in the Dark condition (Table 6), the main effects of the SF and *HD* group are statistically significant, but the interaction between SF and α is not significant. These test results and the chart trends indicate that the sub-band contrast’s effectiveness is stronger in the low *HD* group than in the high *HD* group in the Dark condition, but surprisingly the effective SFs are constant regardless of α.

There is a possibility that the response bias from the first author, HK, who participated in the experiment, accidentally caused the trends in effective SFs because the effects of the sub-band contrast manipulation are not necessarily pronounced. To check this possibility, we reanalyzed the data without the author’s responses in the same way as the regression analysis (Figures 7, 8). We calculated the correlation coefficient between the slopes for the original data and the data without the author. The correlation coefficient was 0.95, indicating that the first author’s bias was unlikely to have caused the trends in effective SFs.

In summary, we found that enhancing the contrast in various SFs increased perceived glossiness in both highlight conditions, although the magnitude of its effect was not very prominent. However, the trends across the surface roughness were different between the highlight conditions. In the Full condition, the effective SF differed across α, as expected, while in the Dark condition where observers were forced to rely on low-luminance regions for glossiness judgment, the most effective SF was always 32 cpi (4.32 cpd) regardless of α. In addition, the effectiveness of SF contrast enhancement was significantly higher in the low *HD* condition than in the high *HD* condition in the Dark condition, but not in the Full condition.

## General Discussion

### Causal Effects of Sub-Band Contrast

In this study, we investigated whether perceived glossiness can be modulated by manipulating the contrast of high spatial frequency (SF). Moreover, we investigated the effectiveness of the spatial frequencies for glossiness manipulation per surface roughness α. The stimuli were object images with different α, and the RMS contrasts of the sub-band images at different spatial frequencies were manipulated as an experimental parameter.

In both highlight conditions, the results demonstrate that glossiness can be enhanced by increasing the RMS contrasts of the sub-band images for most stimulus conditions. In terms of their effect size, perceived glossiness was increased only by approximately 36 points [ = 60 (slope) × 0.6 (increased contrast)] in the best case. The impacts of contrast manipulation on glossiness seems smaller than previous studies that demonstrated the impacts of sub-band contrast on different types of material perception (e.g., Giesel and Zaidi, 2013; Boyadzhiev et al., 2015). Nevertheless, the present results are consistent with the ideas raised in the following previous studies. First, “sharpness” of specular highlights is a cue for glossiness perception (Beck and Prazdny, 1981; Ferwerda et al., 2001; Marlow et al., 2012; Marlow and Anderson, 2013). Second, luminance contrast in high SF bands, such as luminance edges, was also suggested to be a cue for glossiness perception, which exists in low-luminance regions (Kim et al., 2012; Kiyokawa et al., 2019). However, Kiyokawa et al. (2019) showed only the statistical correlations between glossiness and luminance contrast in high SF bands. In contrast, our present findings suggest a causal relationship between them, not only the correlations, although the magnitude of its effect was not very prominent. Namely, our results indicate the importance of contrast in high spatial frequency bands by demonstrating the causal effects of contrast enhancement on glossiness.

### Effective Spatial Frequency

Additionally, we found differences in glossiness enhancement effects across SFs. In the Full condition, SFs most effective for glossiness enhancement differed among the surface roughness; the effective SF for glossiness enhancement was lower for objects with rough surfaces (high α), and higher for objects with smooth surfaces (low α). In short, there was a negative correlation between effective SF and α. This negative correlation is intuitively plausible from the physical property of specular reflections because the specular reflection components should be blurred on rough surfaces with high “αs”.

In contrast, surprisingly, we found little effect of surface roughness on the effective SFs for glossiness enhancement in the Dark condition. Our results showed that perceived glossiness increased in the Dark condition images whose sub-band contrasts were enhanced as well as in the Full condition, but the effective spatial frequency was constant at 32 cpi (4.32 cpd) regardless of the surface roughness. This seems counterintuitive because spatial frequency, which contains rich information about specular reflections, should change with surface roughness directly linked to the degree of specular reflection blur. In the Dark condition, high SF components derived from the specular components should exist only in dark regions. Thus, the differences in the effective SFs between the Full and Dark conditions raise the possibility that the visual system changes the glossiness perception strategy regarding the spatial frequency dependence according to the object surface luminance.

Several possible factors are inducing the constant dependence of glossiness on the middle SF band in the Dark condition. Here, we focus on three candidate factors.

#### (1) Effects of contrast sensitivity properties of luminance vision

One of the candidate factors is the luminance contrast sensitivity properties of the visual system. The spatial contrast sensitivity function (CSF) for luminance vision is well known to exhibit the band-pass characteristic in most environments (Robson, 1966; Campbell and Robson, 1968), although the apparent contrast on suprathreshold stimuli is relatively flat across SFs (Georgeson and Sullivan, 1975). The peak sensitivity of luminance CSF is approximately 3–4 cpd, close to the Dark condition’s most influential SF. Further, the visual system has to detect subtle and near-threshold contrasts when relying on the Dark condition’s sub-band information, because most of the informative sub-band information exists in dark regions. On the contrary, in the Full condition, the images have sufficiently high contrast in the bright regions such as highlights. In this case, observers can rely on highly suprathreshold sub-band contrast, known to be flat across SFs (Georgeson and Sullivan, 1975). Therefore, the relative effectiveness of different SFs may not be significantly affected by the CSFs. Consequently, the visual system may change the strategy for glossiness when relying on dark components considering its contrast sensitivity properties.

Scale-variant properties should give us a hint about the involvement of CSF in the effectiveness of sub-band contrast. If the CSFs caused a constant dependence on a specific SF in glossiness perception, the perception should exhibit scale-variant properties. To casually check if the SF properties of glossiness perception are scale-variant or scale-invariant, we additionally performed the same experiment as Experiment 2, but with a double observation distance (116 cm) for five observers (see Supplementary Figure S5). The results showed that the trends regarding the dependence on SFs were quite similar to those in Experiment 2; the peaks of the regression slopes are found at 32 cpi in the Dark condition. These results raise the possibility that the fixed SF peaks in the Dark condition reflect scale-invariant mechanisms in perceived glossiness. Considering this result, CSF is unlikely to induce a constant dependence on 32 cpi in the Dark condition.

#### (2) Relations to spatial frequency components of our stimulus shapes

Alternatively, “32 cpi” may reflect some features of our object shapes. We calculated the SF amplitudes on the matte object images of the three shapes used for our stimuli to check this possibility. The amplitudes were averaged across orientations. Then, they were compared with those of a matte sphere with no bumpy relief. The difference in the amplitudes between our shapes and a sphere (our shapes–sphere) is shown in Supplementary Figure S6. The differences appear in a wide range of SFs, but not in some specific SFs. Thus, we did not find any evidence that 32 cpi reflects the features of our stimulus shapes.

#### (3) Accidental increase of 32 cpi contrast in creating Dark stimuli

The last possibility is that the 32 cpi sub-band in the Dark condition stimuli picked up more edges than other SFs made when the matte components have replaced the specular components. To test this possibility, we extracted luminance edges in the same way as in Experiment 1. We calculated the ratio of the luminance edge numbers of the Dark condition to the Full condition for each SF. The ratios are summarized in Table 7. The Dark condition stimuli contained more luminance edges than the Full ones in general. In particular, the ratio was highest in the 32 cpi condition. This seems relevant to the highest effectiveness of 32 cpi in Experiment 2. However, these edge numbers are unlikely to be a dominant factor determining the relative efficacy of the SFs, because the differences in the edge number ratios exhibit only subtle differences among the SFs compared to the large difference in the regression slopes.

**TABLE 7:** The difference in the numbers of luminance edge pixels between highlight conditions.

SF	Ratio of edge numbers (Dark/Full)
8 cpi	0.96
16 cpi	1.01
32 cpi	1.13
64 cpi	1.10

In summary, our present findings suggest that the visual system seems to rely on information in a specific SF (32 cpi in our case) for glossiness perception when the perception is based on dark regions. Although 32 cpi corresponds to the peak SF of the CSFs, doubling the viewing distance did not change the effective SFs. Additionally, our object shapes and the manipulations performed for creating the Dark stimuli did not exhibit any unique properties around 32 cpi. These results support the view that some scale-invariant mechanisms, not the scale-variant mechanism such as CSFs, are mainly involved in the dependence of glossiness perception on a specific SF band.

### Remaining Issues

Does the dependence of perceived glossiness on specular highlights affect the sub-band contrast’s contribution to perceived glossiness? Kiyokawa et al. (2019) stated that the number of luminance edges extracted by a Laplacian filter could explain perceived glossiness only for the stimuli on which glossiness perception does not depend on the specular highlights (i.e., low *HD* stimuli). Conversely, in the current experiment, we measured the sub-band contrast enhancement’s impact on perceived glossiness, and found no significant differences in contrast enhancement effectiveness between the *HD* groups in the Full condition (Figure 8A). This apparent conflict is considered to have arisen from the differences in image features used in the analyses, namely, the luminance edges extracted using the Laplacian filter in the previous study and the sub-band contrast extracted using the FIR filters in the current study. In Kiyokawa et al. (2019), high *HD* stimuli typically showed a low specularity (Figure 12 in Kiyokawa et al., 2019). Therefore, edge extraction using the Laplacian zero-cross method based on a certain threshold may have discarded the pale specular-derived edges in high *HD* (low specularity) stimuli. In contrast, because the FIR filter captures all sub-band components without discarding the pale edges, the effects may not have differed across the *HD* groups. However, it is still unclear whether the dependence of perceived glossiness on specular highlights directly affected the impact of high SF components on perceived glossiness from our results.

It is still unclear how our visual system determines the informative SF, which contains rich specular components. Physically, the SF of the specular components is rarely lower than that of the diffuse components because the specular components include the mirrored reflection of the surroundings and therefore have steeper luminance gradients. Thus, one possibility is that our visual system simply relies on comparing the amplitude between relatively higher SFs and lower SFs because this comparison may have information regarding the dominant SFs of the specular components, at least partly. Of course, more sophisticated image analysis, such as an analysis of the orientation field and luminance gradient, should also be involved. Such computational mechanisms need to be elucidated in future studies.

## Data Availability Statement

The raw data supporting the conclusions of this article will be made available by the authors, without undue reservation, to any qualified researcher.

## Ethics Statement

The studies involving human participants were reviewed and approved by the ethical committee of the Faculty of Engineering, Yamagata University. The patients/participants provided their written informed consent to participate in this study.

## Author Contributions

HK, TT, YY, and TN designed the research and analyzed data. HK performed the experiments. HK and TN wrote the manuscript. All authors approved the manuscript for publication.

## Conflict of Interest

The authors declare that the research was conducted in the absence of any commercial or financial relationships that could be construed as a potential conflict of interest.

## Footnotes

1 https://hdrihaven.com
