# Supplementary material for: Spatial Frequency Effective for Increasing Perceived Glossiness by Contrast Enhancement
Source: Front Psychol. 2021 Feb 5;12:625135. doi: 10.3389/fpsyg.2021.625135 (PMC7892470; doi:10.3389/fpsyg.2021.625135)
Supplement: Supplementary file 1 [file Table_1.docx]

Supplementary Material

# Supplementary table

The number of stimuli in each $\alpha$ and *HD* groups is shown in Table S1.

**Table S1**. Number of stimuli in each pairing of $\alpha$ and *HD* group.

| HD group | $\alpha$ | Number of stimuli |
| --- | --- | --- |
| High | 0.01 | 6 |
|  | 0.05 | 10 |
|  | 0.1 | 9 |
| Low | 0.01 | 12 |
|  | 0.05 | 8 |
|  | 0.1 | 9 |

# Supplementary figures

The figure grid showing the effect of specularity and roughness is shown in Figure S1.


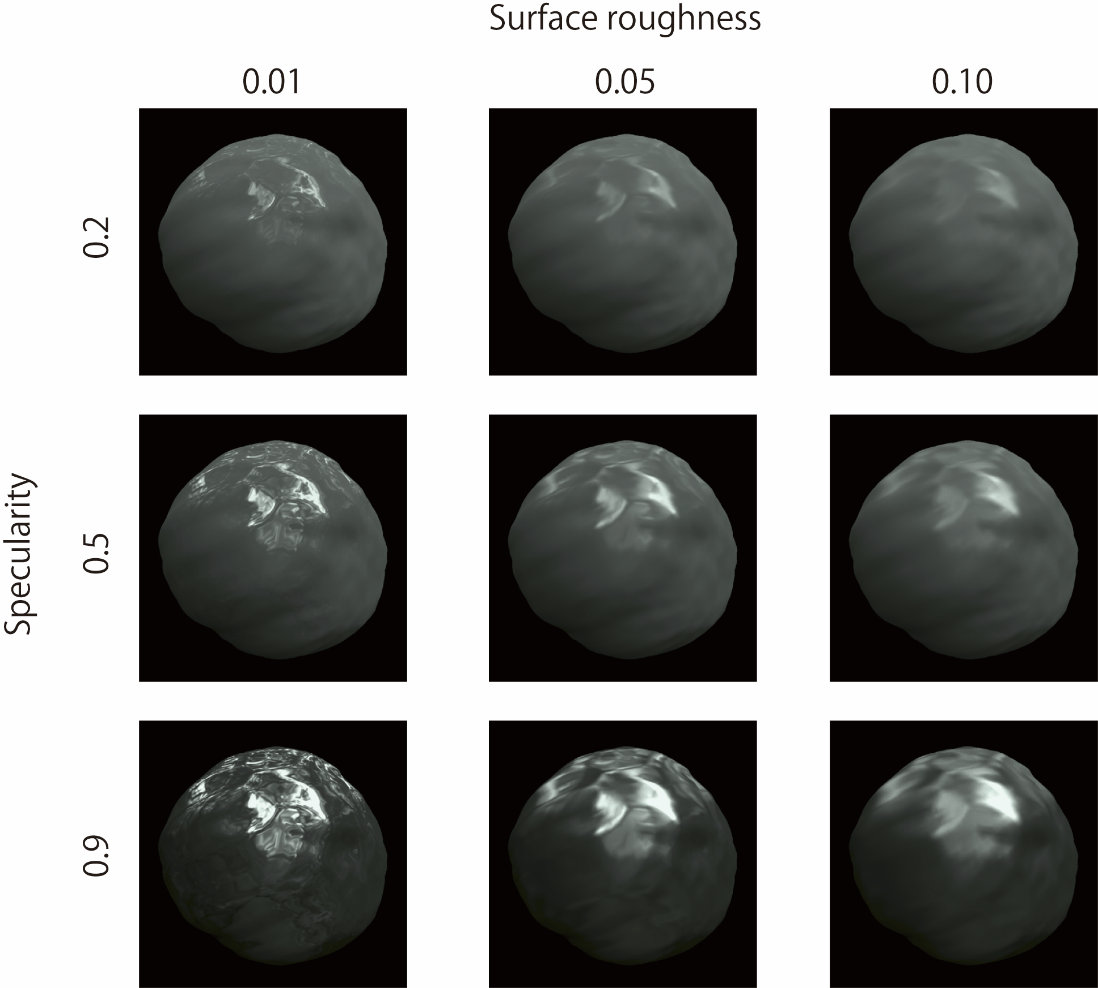


**Figure S1.** Stimulus images with different specularity and roughness.

Example images in which the sub-band contrast is increased at 16, 32, or 64 cpi are shown in Figure S2–S4.


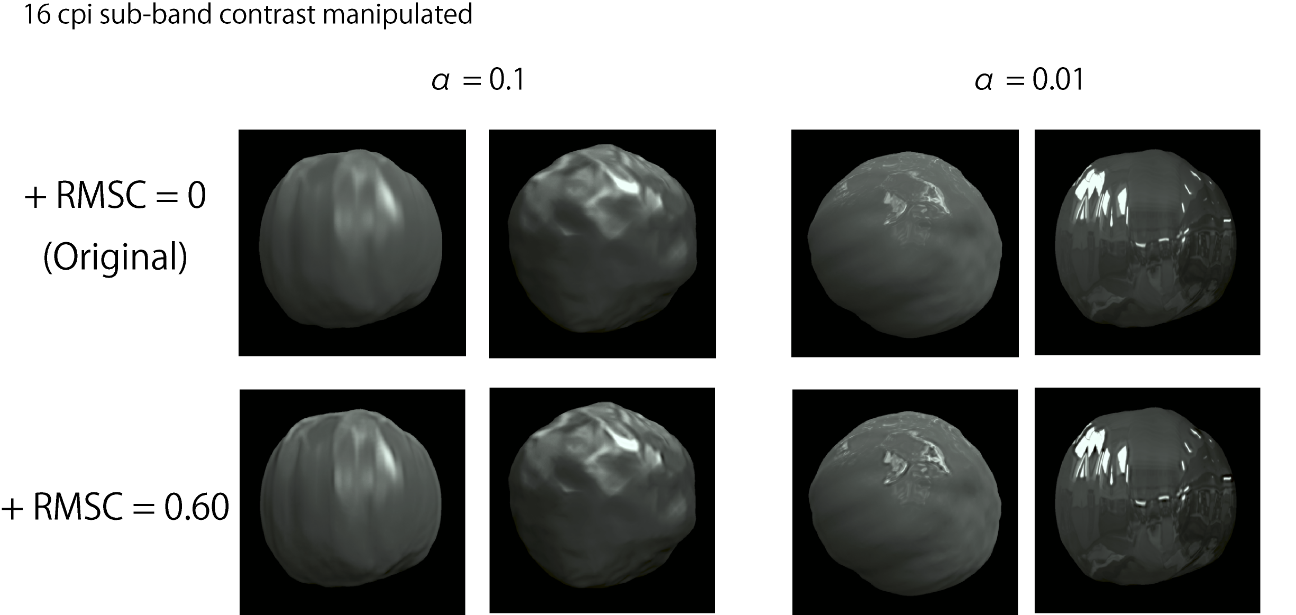


**Figure S2.** Example of stimuli with enhanced contrast in 16 cpi.


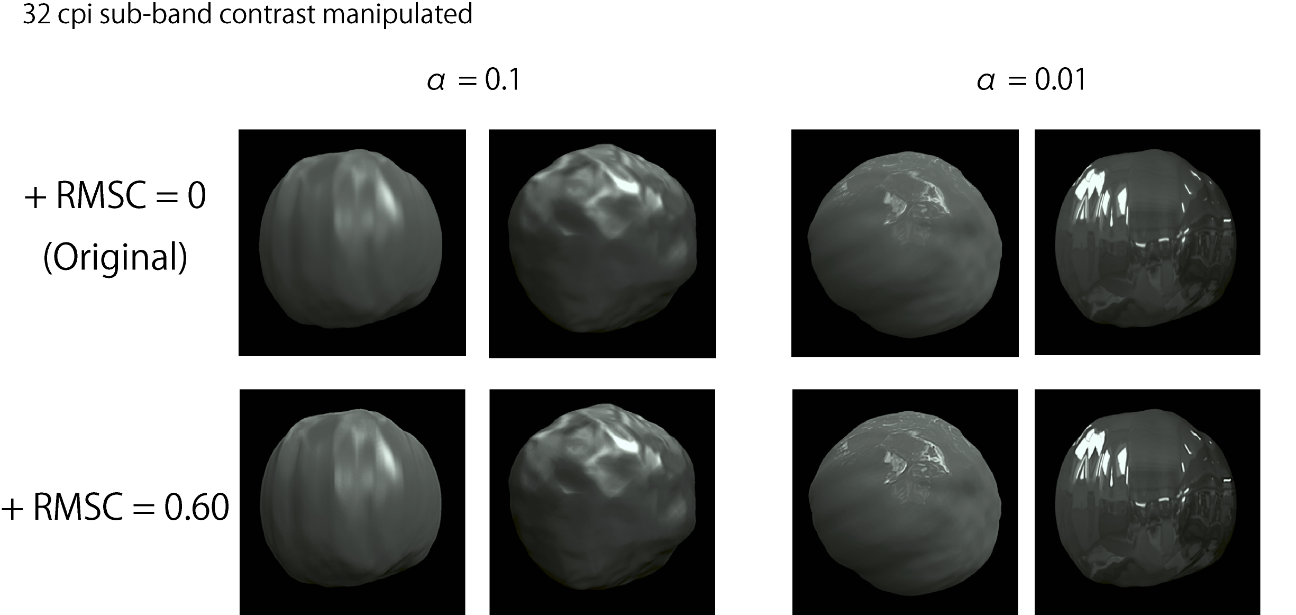


**Figure S3.** Example of stimuli with enhanced contrast in 32 cpi.


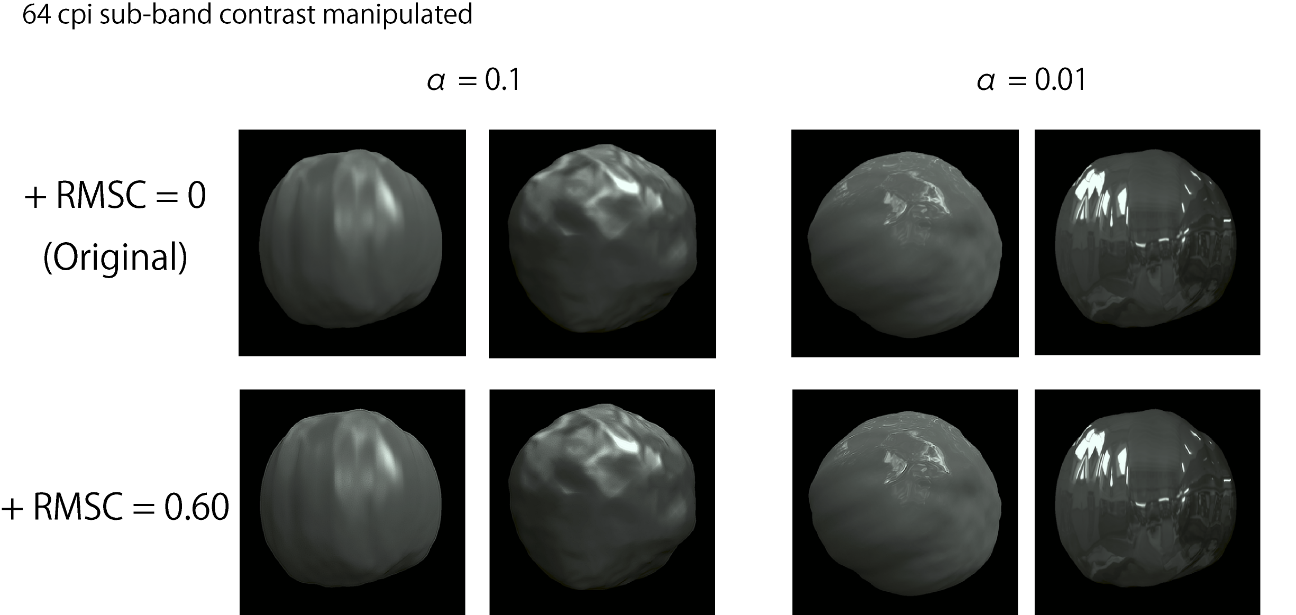


**Figure S4.** Example of stimuli with enhanced contrast in 64 cpi.

The results of the experiment that we additionally performed the same experiment as Experiment 2, but with a double observation distance (116 cm) are shown in Figure S5.


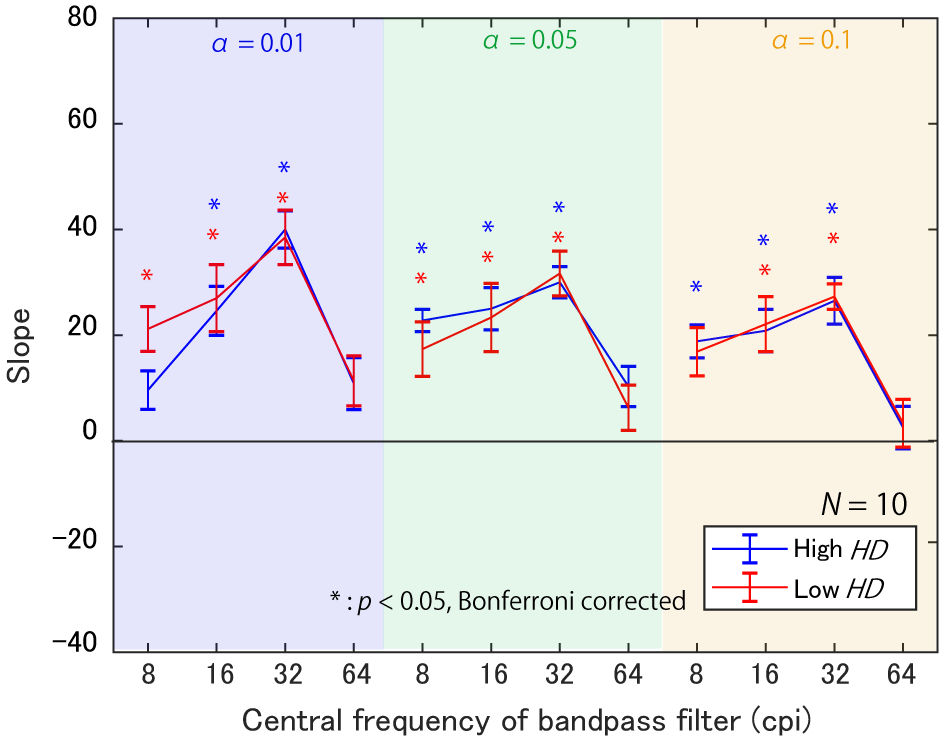


**Figure S5.** The slope of regression lines between contrast enhancement strength and selection probability in the additional experiment (see General discussion in the main text).

The results of difference in the amplitudes between our shapes and a sphere (our shapes–sphere) are shown in Figure S6.


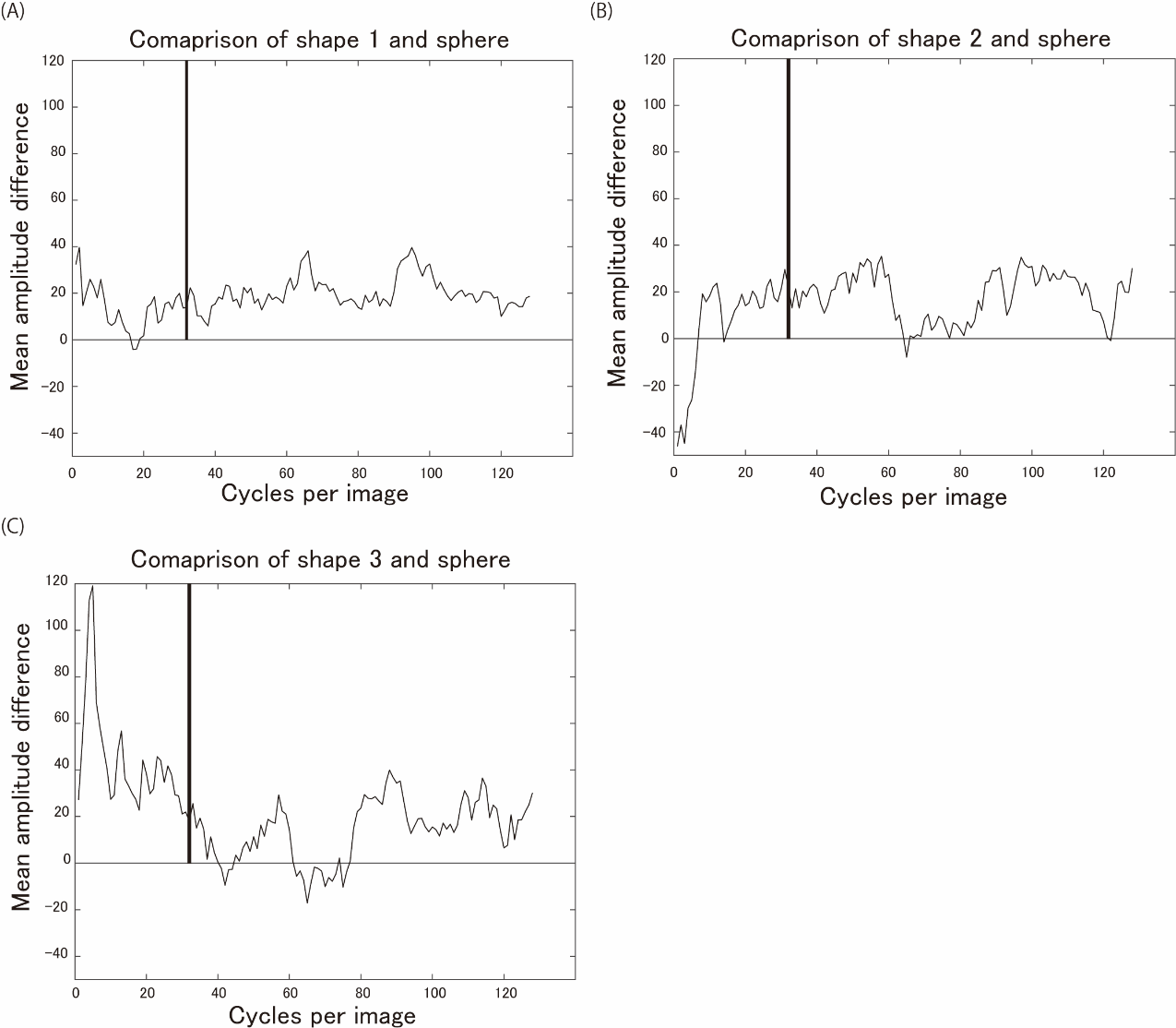

**Figure S6.** The difference in SF amplitudes between images of our three shapes used and a sphere. The bold bar shows the position of 32 cpi.
